# Editorial: Mesenchymal and induced-pluripotent stem cells as models to study biological processes

**DOI:** 10.3389/fgene.2024.1439306

**Published:** 2024-07-25

**Authors:** Melvin A. Ambele, Catarina O. Miranda

**Affiliations:** ^1^ Department of Immunology, Institute for Cellular and Molecular Medicine, SAMRC Extramural Unit for Stem Cell Research and Therapy, Faculty of Health Sciences, University of Pretoria, Pretoria, South Africa; ^2^ Department of Oral and Maxillofacial Pathology, School of Dentistry, Faculty of Health Sciences, University of Pretoria, Pretoria, South Africa; ^3^ CNC—Center for Neuroscience and Cell Biology, University of Coimbra, Coimbra, Portugal; ^4^ CIBB- Center for Innovative Biomedicine and Biotechnology, University of Coimbra, Coimbra, Portugal; ^5^ III—Institute for Interdisciplinary Research, University of Coimbra, Coimbra, Portugal; ^6^ GeneT, Center for Excellence in Gene Therapy in Portugal, University of Coimbra, Coimbra, Portugal

**Keywords:** mesenchymal stromal/stem cells, induced-pluripotent stem cells, organoids, disease models, biological processes

Mesenchymal stromal/stem cells (MSCs) are isolated directly from various body tissues and are known for their capacity to self-renew, multipotent lineage differentiation and the secretion of bioactive factors, making them suitable for potential use in tissue repairs, regenerative medicine and as cellular therapy (Oliveira Miranda et al., 2018; Barros et al., 2020; Jovic et al., 2022; Ghasemi et al., 2023; Li et al., 2024; Sitbon et al., 2024). Additionally, MSCs can be used as cellular models for understanding biological processes (Visweswaran et al., 2015; Ambele et al., 2016; Jiang et al., 2019; Liang et al., 2022). On the other hand, induced pluripotent stem cells (iPSCs), are adult somatic cells that have been genetically reprogrammed by inducing pluripotency similar to embryonic stem cells. iPSCs posses unlimited self–renewal capacity and can be induced to differentiate into all adult cell types, thereby making them a unique model for studying a variety of biological processes (Yin et al., 2024). Together, current research trend puts MSCs and iPSCs as promising tools at the forefront of groundbreaking research that seeks to explore cellular therapy as alternative treatment for various conditions or for use in mimicking the *in vivo* physiological environment to provide better understanding of the pathophysiological processes of disease.

**FIGURE 1:**
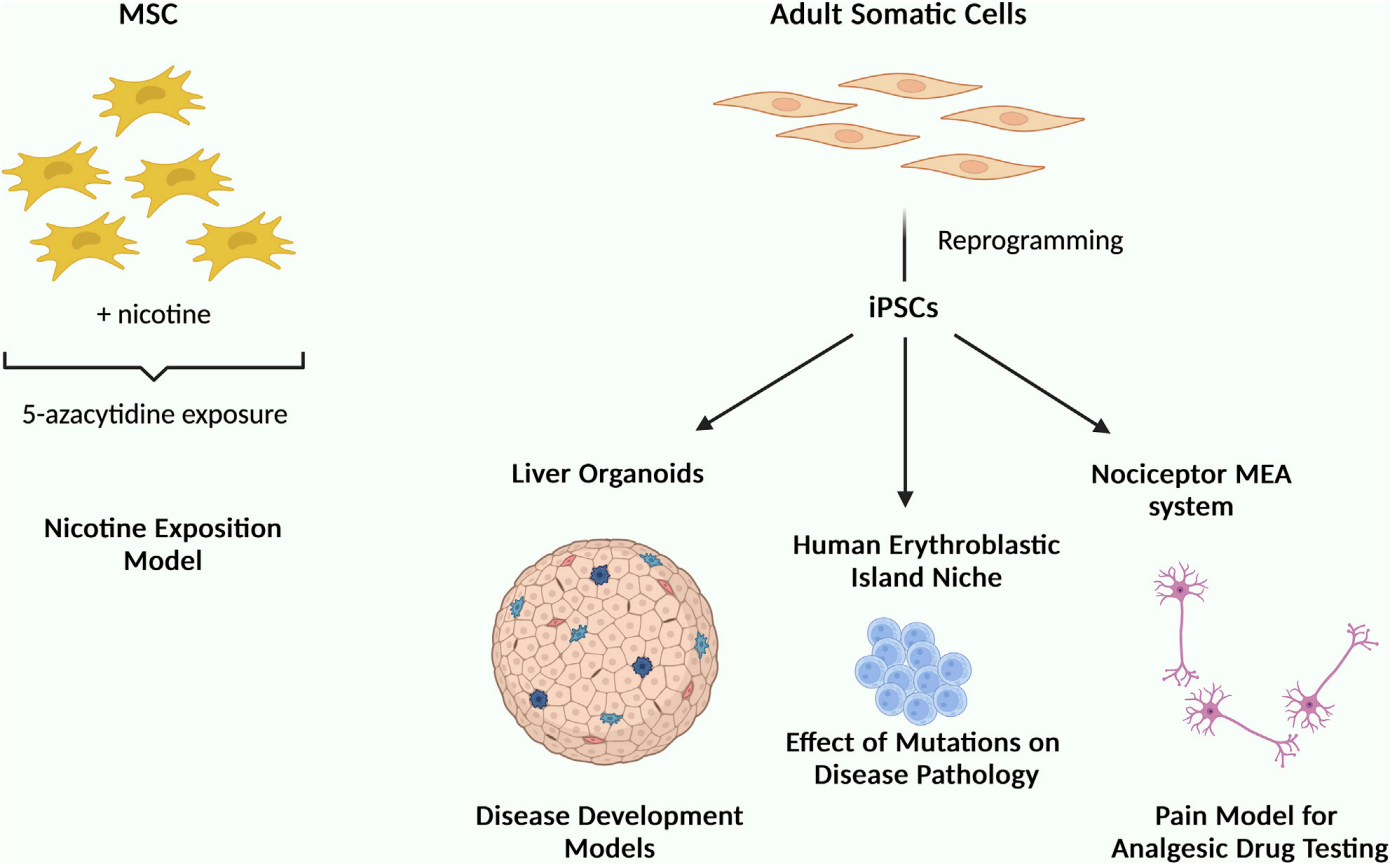
Schematic illustration summarizing the studies of this Research Topic “*Mesenchymal and Induced-Pluripotent Stem Cells as Models to Study Biological Processes*” showing the use of MSCs and iPSCs as cellular models to understand disease mechanisms and for drug screening.

Research over the years have extensively explored the potential of MSCs as cellular models to study biological processes such as adipogenesis (Ambele et al., 2016), osteogenesis (Jiang et al., 2019) and chondrogenesis (Liang et al., 2022), as well as cellular therapy in treating different conditions globally (Jovic et al., 2022) by paracrine mechanism. This Research Topic brings together current works that uses MSCs and iPSCs to provide new insights on 1) the development of biological systems, and 2) the understanding of molecular and cellular processes as illustrated in Figure 1.

In one of these studies, MSCs were exposed to nicotine and then treated with 5-azacytidine to mimic the risk of smoking on cells expressing the cardiac markers GATA4 and troponin (Figure 1). The authors reported nicotine reduced cells viability and the expression of these markers (Gheisari et al.). As has been previously reported in many other studies, this study highlights the potential use of MSC in understanding molecular process.

Regarding iPSC, a study showed that iPSCs-derived nociceptor culture system integrated with microelectrode arrays could be used for nociceptive analgesic drug testing. This is particularly important given the global health crisis in opioid abuse and addiction for chronic pain management and that this kind of studies are undesirable to be performed in animals and/or humans. This system offers alternative strategy to search for new effective analgesics and opioid substitutes (Nimbalkar et al.). Generally, one of the advantages of using iPSCs is the ability to produce cellular models that resemble human disorders as well as to generate organoids that allow cells to assembled in a 3D configuration resembling the tissue structure *in vivo*. For example, iPSC-derived liver organoids can serve as useful tool to study organogenesis, pathological modeling through cell-cell interactions, disease modeling and drug screening (Ouchi and Koike). One study used iPSCs to recapitulate the human erythroblastic island (EBI) niche in congenital dyserythropoietic anaemia (CDA) type IV to study the effect of KLF1 mutation Glu325Lys (E325K) on the disease pathology. The authors reported this mutation to negatively affect the production of erythroid cells. Also, upon expression of this mutated gene KLF1-E325K, a slight reduction of both RBC enucleation and macrophage maturation were observed. Furthermore, this provides an effective strategy to study the effects of other KLF1 mutations on EBI niche (May et al.).

Notwithstanding, the potential of iPSCs is enormous extending beyond disease modeling and drug screening to encompass cellular therapy and biomarkers discovery, although there is limited information regarding their safety for use as cellular therapies (Yin et al., 2024).

In summary, this Research Topic highlights the use of both MSCs and iPSCs as cellular models, providing new insights into different fields of research and evidence of their potential to study molecular and cellular processes.
